# Diseases of Children

**Published:** 1905-04-29

**Authors:** 


### DISEASES OF CHILDREN.

(*Continued from page* 75.)

**Convulsive Tic.**—"In the main," writes Dr. H. T. Patrick of Chicago,[9] "convulsive tic may be said to be a habit spasm, but it is more than a habit, less than a spasm. Strictly speaking, it is no spasm at all. It is a movement based on an uncontrolled, and, except for a brief space, uncontrollable impulsion." This is the fully developed condition. He believes that in its inception a tic is always voluntary, and that it is only by repetition that it becomes automatic. He illustrates the commencement of the affection as follows. A child has an ill-fitting collar, or a coat too tight in the arm-hole. This begets a twisting or drawing of the neck, with tilting of the chin, or a writhing shrug of the shoulder. The collar is removed, the arm-hole enlarged, but the abnormal movement continues. It must be noted that not every child will develop tic under these conditions, but only those who are specially sensitive to sensations, and more readily translate a sensation into motion. The subjects of tic are too sensitive, too impressionable, their sensori-motor mechanism is too active, and they lack instinctive as well as voluntary inhibition. The movements in tic, unlike those in chorea, are but repetitions of the same act. They are purposive, although one may not be able to trace the original purpose, and as such are in marked contrast to the inco-ordinate jerkings and writhings of chorea, the vermicular twistings of athetosis, and the rhythmic oscillations of hysteria. In course of time the movements may spread from the muscles originally affected, at first locally only, but later the whole body may be involved. Convulsive tic can be voluntarily restrained to some extent, and in many cases can be quite controlled for a time by some manœuvre which the patient has instinctly or deliberately hit on. The prognosis is ordinarily good, and most children recover without any special treatment. Some, however, retain the habit up to and through adult life, and when this is so the location usually changes—for instance, a tic about the nose changing into lifting the eye-brows.

**Recurrent or Periodic Vomiting.**—Cases of this somewhat uncommon condition have been recorded by Batty Shaw and Tribe [10] and by F. Langmead.[11] An attack of vomiting comes on suddenly, without any premonitory symptoms of ill-health, the vomited matter being at first food, later mucus, and perhaps bile and traces of blood. An attack usually lasts from a few hours up to 24 hours, but occasionally it is prolonged for a fortnight. Retching, thirst, loss of appetite, prostration, and rapid wasting are usually present. The temperature and pulse are normal, save in exceptional cases, and rapid recovery follows the vomiting attack, except when it is prolonged. Treatment during an attack is of little benefit, and between the attacks there are few symptoms which call for any treatment. The etiology of these cases is very obscure. Langmead lays stress on a hereditary neurotic tendency, and all writers are agreed as to this. Rachford attributes the vomiting to an autointoxication due to incompetence of the liver. This may be called the "lithæmic" theory, and he lays stress on the association with migraine, which had supervened in four of his cases. The recognition of acetone in the breath, urine, and vomit, by Marfan has led him to believe that acetone is the toxic factor and that the condition is one of acetonæmia. Edsall describes the affection as an "acidosis" or "acid-intoxication." By this term is meant the reduction of the alkalinity of the fluids of the body by the intervention of acid bodies. The strongest support in favour of this view is the marked benefit which has followed in some cases from the employment of bicarbonate of soda in large doses. Shaw and Tribe's patient was a girl of 11 years, who had suffered since the age of three years from recurrent vomiting, seven attacks in all coming on without any apparent cause or warning. She was extremely emaciated, weighing only 1 st. 10½ lbs. The alkaline method of treatment was of no benefit, but recovery followed on rest and careful feeding. Langmead's first case was that of a girl, age not stated, who was admitted into hospital in a fifth attack of recurrent vomiting, and sank rapidly into a state of convulsions and coma, very similar to that associated with diabetes. At the post-mortem examination the only definite pathological change was advanced fatty degeneration of the liver. The second patient, aged six and a-half years, had already suffered from 10 or 12 attacks of vomiting, dating from infancy. He had been suffering for four days from vomiting, irritability, restlessness, and drowsiness. On admission he was semi-conscious, with flushed face and sunken eyes. There was double ptosis. Acetone could be detected in the breath, and diacetic acid in the urine. Two days later he was convalescent. No specific treatment was adopted in this case. The above extracts show that this recurrent vomiting of children deserves more study than it has hitherto received, for it may prove fatal in exceptional cases, and at present we are entirely at a loss as to the etiology, pathology, and treatment.

**Lumbar Puncture.**—The value of lumbar puncture in the diagnosis of disease is now well established, although it has not achieved so much from a therapeutic point of view as was at one time expected. The exact technique of the operation is fully described by Dr. Purves Stewart.[12] The best spot to tap the arachnoid sac is between the laminæ of the fourth and fifth lumbar vertebræ. A transverse line drawn between the summits of the iliac crests will be found to cross the vertebral column at the tip of the fourth lumbar spine. The needle is to be inserted immediately below this spot. The skin having been disinfected and the exploring syringe sterilised, a spray of ethyl chloride should be used to freeze the site of puncture. The needle is then inserted below the fourth spinous process forwards through skin, subcutaneous tissues, and ligaments until the ligamentum subflavum is reached and is recognised as more resistant. Pushing on through this, the needle enters the arachnoid sac. If bone is struck, the needle is partly withdrawn and again pushed inwards at a slightly higher or lower level. The depth at which the sac is reached is in children from 1 to 1½ inches. Under ordinary conditions the fluid will ooze steadily, drop by drop, but when it is under tension there will be a steady flow at first or even a rush of fluid. Examination of the fluid, bacteriologically and microscopically, will often give valuable information as to the form of meningitis present, whether it is tuberculous, or pneumococcal, or cerebro-spinal.

[9] Jour. Amer. Med. Assoc., Feb. 11, 1905. [10] Brit. Med. Jour., Feb. 18, 1905. [11] Ibid. [12] Internat. Clin., Vol. iii., 14th series.

